# Lucanthone and Its Derivative Hycanthone Inhibit Apurinic Endonuclease-1 (APE1) by Direct Protein Binding

**DOI:** 10.1371/journal.pone.0023679

**Published:** 2011-09-15

**Authors:** Mamta D. Naidu, Rakhi Agarwal, Louis A. Pena, Luis Cunha, Mihaly Mezei, Min Shen, David M. Wilson, Yuan Liu, Zina Sanchez, Pankaj Chaudhary, Samuel H. Wilson, Michael J. Waring

**Affiliations:** 1 Biology Department, Brookhaven National Laboratory, Upton, New York, United States of America; 2 Medical Department, Brookhaven National Laboratory, Upton, New York, United States of America; 3 Department of Genetics and Genomic Sciences, Mount Sinai School of Medicine, New York, New York, United States of America; 4 Department of Structural and Chemical Biology, Mount Sinai School of Medicine, New York, New York, United States of America; 5 NIH Chemical Genomics Center, National Institutes of Health, Rockville, Maryland, United States of America; 6 Laboratory of Molecular Gerontology, Biomedical Research Center, National Institute on Aging, National Institutes of Health (NIH), Baltimore, Maryland, United States of America; 7 Laboratory of Structural Biology, National Institute of Environmental Health Sciences, National Institutes of Health (NIH), Research Triangle Park, North Carolina, United States of America; 8 Undergraduate Biology, Stony Brook University, Stony Brook, New York, United States of America; 9 Department of Pharmacology, University of Cambridge, Cambridge, United Kindgom; University Paris Diderot-Paris 7, France

## Abstract

Lucanthone and hycanthone are thioxanthenone DNA intercalators used in the 1980s as antitumor agents. Lucanthone is in Phase I clinical trial, whereas hycanthone was pulled out of Phase II trials. Their potential mechanism of action includes DNA intercalation, inhibition of nucleic acid biosyntheses, and inhibition of enzymes like topoisomerases and the dual function base excision repair enzyme apurinic endonuclease 1 (APE1). Lucanthone inhibits the endonuclease activity of APE1, without affecting its redox activity. Our goal was to decipher the precise mechanism of APE1 inhibition as a prerequisite towards development of improved therapeutics that can counteract higher APE1 activity often seen in tumors. The IC_50_ values for inhibition of APE1 incision of depurinated plasmid DNA by lucanthone and hycanthone were 5 µM and 80 nM, respectively. The K_D_ values (affinity constants) for APE1, as determined by BIACORE binding studies, were 89 nM for lucanthone/10 nM for hycanthone. APE1 structures reveal a hydrophobic pocket where hydrophobic small molecules like thioxanthenones can bind, and our modeling studies confirmed such docking. Circular dichroism spectra uncovered change in the helical structure of APE1 in the presence of lucanthone/hycanthone, and notably, this effect was decreased (Phe266Ala or Phe266Cys or Trp280Leu) or abolished (Phe266Ala/Trp280Ala) when hydrophobic site mutants were employed. Reduced inhibition by lucanthone of the diminished endonuclease activity of hydrophobic mutant proteins (as compared to wild type APE1) supports that binding of lucanthone to the hydrophobic pocket dictates APE1 inhibition. The DNA binding capacity of APE1 was marginally inhibited by lucanthone, and not at all by hycanthone, supporting our hypothesis that thioxanthenones inhibit APE1, predominantly, by direct interaction. Finally, lucanthone-induced degradation was drastically reduced in the presence of short and long lived free radical scavengers, e.g., TRIS and DMSO, suggesting that the mechanism of APE1 breakdown may involve free radical-induced peptide bond cleavage.

## Introduction

APE1 (also termed Ref-1, APEX, HAP1, AP endo) is a multifunctional protein with distinct activities assigned to different parts of its structure. The N-terminal region is responsible for its redox function, whereas the endonuclease activity is mediated by the larger C-terminal portion [1]–[3]. APE1 is abundant in human cells and accounts for nearly all of the apurinic/apyrimidinic (AP) site cleavage activity found in cellular extracts [4]. APE1 has a strong Mg^2+^ -dependent AP endonuclease activity, a 3′-phosphodiesterase activity, a 3′-mismatch exonuclease activity, and in addition to its DNA repair functions, a redox activity whereby it can reduce a conserved cysteine residue in a target transcription factor, e.g. AP-1 (Jun/Fos), to activate cognate DNA binding. APE1 also stimulates the sequence-specific DNA binding activities of HIFα, NF_κ_B, Pax5, Pax8, Myb and related activating transcription factor/cAMP – responsive element binding proteins [5].

APE1 incision activity is altered in response to radiation and chemotherapy in medulloblastoma and primitive neuroectodermal tumors [6]. Silber *et al*. also showed that APE1 repair activity, which is increased by oxidative stress, contributes to resistance of human glioma cells to alkylating agents [7]. Human glioma cell lines that show lower APE1 expression were more sensitive to methyl methanesulfonate (MMS) and H_2_O_2_, known inducers of AP sites and single strand breaks in DNA [8]. Robertson *et al* [9] have shown that over-expression of APE1 in NT2 cells confers resistance to bleomycin and radiation. Recently, we demonstrated a correlation between APE1 and radiation sensitivity with glioma cell culture models [10]. When APE1 was over-expressed in U251 cells, they became more radioresistant contingent on the level of APE1 over-expression, whereas siRNA depletion of APE1 was associated with radiation sensitivity. This correlation was reiterated by recent studies where APE1 siRNA down-regulation in either colorectal tumor cells *in vitro* or in a subcutaneous nude mouse colon cancer model enhanced radiosensitivity as revealed by increased apoptosis [11]. In addition to the siRNA studies, we modulated APE1 repair nuclease function using two of its known small molecule inhibitors, lucanthone (1-[2-diethylaminoethylamino]-4-methylthioxanthen-9-one) [12] and CRT0044876 (7-Nitroindole-2-carboxylic acid) [13], and showed that APE1 inhibition resulted in increased radiosensitivity.

Due to the dual function of APE1, several inhibitors are being discovered which selectively inhibit either its DNA repair or redox function. The DNA repair inhibitors include the indirect inhibitor methoxamine (MX) [5], [14] and the direct/indirect inhibitors such as lucanthone and CRT0044876. The redox function (Ref-1) inhibitors are soy isoflavones [15], E3330 [16]–[17] and its benzoquinone and naphthoquinone analogues [18], PRNI-299 [19], BQP [20] and resveratrol [21]. Thus, recent efforts have focused on the potential to strategically regulate APE1 protein activity in cells, possibly through the use of small molecular inhibitors, as a means of improving therapeutic agent response.

Lucanthone (CAS479-50-5) and hycanthone (CAS3105-97-3) belong to a family of thioxanthenones and were originally synthesized for use as anti-schistosomal drugs. They were also determined to be DNA intercalators, and like actinomycin D, inhibited RNA synthesis as well as the DNA processing enzymes topoisomerases I and II [22]. The effects of lucanthone are thought to be mediated by its bioactive metabolite, hycanthone [23]. Hycanthone was shown to be a better anti-schistosomal [24] agent than lucanthone. However, due to the negative side effects of hycanthone, including acute hepatic necrosis [25], strong mutagenicity [26] and weak carcinogenicity [27], the use of hycanthone for treatment of human schistosomiasis has been discontinued. Lucanthone, on the other hand, has been used to treat schistosomiasis for almost 20 years before being replaced by new drugs. Work by Turner *et al* [28] showed that radiolabeled lucanthone was more concentrated in neoplastic tissue relative to the surrounding muscle and skin. Since lucanthone is able to cross the blood brain barrier and inhibit cell proliferation without affecting normal non-cycling cells, the compound has been used as an adjuvant for brain tumor radiotherapy [29] and is currently in clinical trial.

Lucanthone is known to inhibit APE1 AP endonuclease activity, without affecting its redox function [12]. Lucanthone is also known to promote accumulation of AP sites in HeLa cells [30], lesions that are substrates for APE1. Since Bailly *et al* [31] showed that both lucanthone and hycanthone preferentially intercalate at AT-rich sequences in DNA, APE1 may be prevented from accessing the AP site due to the presence of DNA-bound lucanthone/hycanthone. Alternatively, lucanthone may elicit its inhibitory effect on APE1 incision activity via direct binding to the protein. As previously reported APE1 structures (PDB ID: 2ISI, 1DEW and 1DE9) show the presence of a hydrophobic site lined by Phe266, Trp280 and Leu282, overlapping the active site of the protein, we hypothesized that hydrophobic molecules like lucanthone/hycanthone would bind at these residues. In addition, based on past evidence [32], we postulated that lucanthone/hycanthone may induce protein oxidation due to the binding capacity and other features of the compound. Data are presented here in support of the idea that lucanthone and its structural analogue hycanthone show very little, if any, inhibition of the DNA (depurinated) binding capacity of APE1 and can indeed predominantly inhibit APE1 endonuclease activity by direct binding to the hydrophobic site and inducing cleavage of the protein via oxidative damage.

## Materials and Methods

### Reagents

The U251-MG glioblastoma multiforme (GBM) cell line was a kind gift from Dr. Dennis Deen of UCSF. These cells were maintained in Eagle's Minimal Essential Medium with 2 mM L-glutamine and 1.5 g/L sodium bicarbonate, supplemented with 10% fetal bovine serum (FBS), and sub-cultured twice a week (1∶3). Cell culture media and FBS were obtained from Invitrogen (Carlsbad, CA). APE1 protein was detected using polyclonal anti-APE1 (Santa Cruz Biotechnology, Santa Cruz, CA) and tubulin was detected using polyclonal anti-©-tubulin (Sigma-Aldrich, St. Louis, MO). ECL kit from Invitrogen has anti-mouse and anti-rabbit –HRP conjugated secondary antibodes which were used at 1∶ 30,000 dilution. Lucanthone and hycanthone (Figure S1) obtained (in 1970s by Michael Waring) from Dr S. Archer, Sterling-Winthrop Research Institute, Rensselaer, NY, were maintained at 4C under hygroscopic conditions, and were dissolved in sterile double distilled water just prior to reactions. Plasmids consisting of full length APE-1 in pET15b and pCMV10 were kind gifts from Dr. T. Izumi and Dr. Hua Fung, of Louisiana State Health Center, New Orleans, LA and Harvard Medical School, Boston, respectively.

### Cells extract preparation

0.5–1.5×10^6^ cells were resuspended in 200 µl of ice-cold 1× cell extraction buffer (50 mM Tris-HCl pH 7.5, 1 mM EDTA, 100 mM NaCl and 1 mM PMSF) and sonicated for 5–10 s in a 4°C bath sonicator (Sonifier Cell Disruptor, Plainview, NY) at a setting of 20 mHz. The sonicates were centrifuged at 10,000 rpm for 5 min at 4°C. Soluble and insoluble fractions were collected (the insoluble pellet was resuspended in 200 µl of cell extraction buffer) and assayed for APE1 protein and enzyme activity as described below.

### Expression and purification of full length APE1

E.coli BL21/DE3 were transformed with the pET15b plasmid containing full length APE1 and these bacterial cultures (500 ml YTB medium) were grown to OD_600_ of 0.6 and the full length APE1 protein was successfully expressed and purified to 25–30 mg protein/L (20 mM HEPES, 200 mM NaCl buffer pH 7.5) culture according to method of Agarwal *et al* [33]. Additional stocks of wild-type, full length APE1, and the APE1 mutant proteins (e.g. F266A), were generated as described [34]. A pCMV-APE1 plasmid [19] was transfected into U251 cells using Lipofectamine 2000 as per manufacturer's instructions followed by selection in G418 as detailed in our recent paper [10] and clones selected for APE1 overexpression.

### SDS-PAGE and Western blot

Total purified protein concentration was determined by the ratio of A_280_/A_260_. 250 ng of APE1 treated with lucanthone or hycanthone (0.05–100 µM) for 2 h at 37°C in final volume of 30 µl were mixed with an equal volume of gel loading buffer (0.001% bromophenol blue, 4% SDS, 10% 2-ME, 20% glycerol, and 125 mM Tris pH 6.8) and denatured at 95°C for 5 min. Total protein concentration in cell extracts was determined using the Bradford assay (BioRad, Hercules, CA) and 25 µg of total protein from soluble or insoluble cell fractions were mixed with an equal volume of gel loading buffer (0.001% bromophenol blue, 4% SDS, 10% 2-ME, 20% glycerol, and 125 mM Tris pH 6.8) and denatured at 95°C for 5 min. This mixture was separated by SDS-PAGE (4% stacking, 7.5% resolving gel) for 2–3 h at 40 mA on a BioRad MiniPROTEAN II Electrophoresis Cell. To detect proteins, proteins were transferred onto a trans-Blot nitrocellulose membrane (0.45 µm; BioRad) overnight at 4°C at 15 mA in standard Tris-Glycine buffer containing 20% ethanol. Membranes were probed using polyclonal anti-APE1 or polyclonal anti-©-tubulin at 1/1,000 dilution in TTBS (0.1% Tween 20 in TBS (pH 7.5)) with secondary, anti-rabbit HRP-conjugated antibody used at 1/30,000 dilution to detect APE1 and ©-tubulin protein control. Chemi-luminescence was developed using an ECL kit according to the manufacturer's instructions and detected by exposing the blot to HyperfilmECL for 30 s–3 min. (GE Healthcare Biosciences, Piscataway, NJ).

### APE1 Endonuclease activity

APE1 endonuclease activity was determined using an assay that measures the conversion of plasmid DNA from supercoiled to relaxed form by incision at an abasic site [35]–[36]. Briefly, the substrate used was 200 ng of depurinated pUC18 DNA in 10 µl of 1× APE1 buffer containing 50 mM Hepes, pH 7.4, 150 mM KCl, 5 mM MgCl_2_ and 100 µg/ml of BSA in presence of different concentrations of the cell extract (1 µl containing 0–55 ng of total protein). Similar reactions mixtures were set up with untreated pUC18, which served as the internal control. This reaction mixture was incubated at 37°C for 15 min, and the reaction was stopped by addition of alkaline stop mix (0.25% bromocresol green in 0.25N NaOH, 50% glycerol) and left at room temperature for 10–15 min. Then the products were resolved on a 0.8% agarose gel in 40 mM Tris-acetate and 2 mM EDTA for 2 h. The gel was stained with ethidium bromide to visualize supercoiled and relaxed plasmid DNA and imaged with a digital imaging system [37] and the area under the supercoiled and relaxed form was determined. Final calculations were done in femtomoles (fmol) of abasic sites incised/min/mg protein with normalization done using Pyruvate Kinase units (PKU) present in these extracts. Poisson distribution calculations were done on supercoiled and relaxed bands to estimate incisions per plasmid molecule and the resulting depurinated pUC18 had 1 AP site per molecule. The endonuclease activity inhibition by lucanthone was analyzed using standard Lineweaver-Burke Plot to determine if the inhibition was competitive or non-competitive.

### Cleavage of APE1 by lucanthone and CRT0044876

Western blotting was carried out by 7.5% SDS-PAGE of cell extracts (20 µg total protein per lane) either from APE1 overexpresser clone 5 pretreated with 2.5–200 µM concentration of lucanthone/CRT0044876 or recombinant APE1 for 2 h at 37°C in presence of protease inhibitor cocktail (2 tablets (Roche, # 11836153001) containing mixture of several protease inhibitors with broad inhibitory specificity for serine, cysteine and metalloproteases in all systems, dissolved in 20 ml of APE1 buffer). CRT0044876 was used as another APE1 small molecule inhibitor with possible direct interaction between itself and APE1 [13]. Pretreatment of Ape1 overexpressor clone 5 cultures with 10 µg/ml of cycloheximide (CHX) for 4 h prior to lucanthone/hycanthone addition, was carried out to determine if these thioxanthenones affected Ape1 protein synthesis. Radioquenchers like 10 mM TRIS, ascorbic acid (100 µM), N-acetyl cysteine (100 µM) (with recombinant APE1) or 1% DMSO (with cell extracts) were used to inhibit the cleavage reactions.

### Direct binding of APE1 to lucanthone/hycanthone

APE1 (and its hydrophobic mutants) and lucanthone/hycanthone reaction stoichiometry was studied using surface plasmon resonance on a BIACORE 2000 apparatus (Biacore, GE Healthcare, Sweden) at the protein core facility in SUNYSB, to determine the affinity of the two drugs for APE1 (or NΔ40 APE1). APE1 and its mutants (10 µg) (RU_max_ values were 9000 RU units after immobilization) were immobilized on a CM5 chip by amine coupling (as per the manufacturer's instructions) and binding experiments were performed at 20°C in 10 mM HBS-EP buffer, pH 7.4. Lucanthone (or hycanthone) (20–700 µM) was injected as analyte over the sensor chip in HBS-EP buffer at 10 µl/min. The regeneration was achieved by 10 mM glycine-HCl, pH 3.0 between each analyte (drug) concentration.

### Circular Dichroism

APE1 and its mutant proteins (10 mg/ml), 50 µl (500 µg) (14 µM) in APE1 buffer (50 mM HEPES, pH 7.4, 150 mM KCl, 5 mM MgCl_2_), were mixed with lucanthone/hycanthone (1 mg/ml), 50 µl (50 µg), 140 µM and incubated at 37°C for 60 min and far UV-CD spectra were recorded at NSLS U11 beam line at BNL. Lucanthone and hycanthone were also scanned as drug controls. The scanning parameters used were a 0.001 cm path length quartz cell, scanning wavelengths from 260 to 170 nm, bandwidth of 0.5 nm, digital integration time of 1 s, time constant of 200 ms, step size of 1 nm, and sensitivity of 200 µV. The data were corrected with blank subtraction from APE1 buffer alone. The secondary structure for APE1 with or without lucanthone or hycanthone was analyzed using CDSSTR program DICHROWEB [38].

### MALDI-TOF

100 nM of APE1 protein was treated with 100 µM of lucanthone, hycanthone or CRT at 37°C for 2 h and 24 h and analyzed on sinipinic acid matrix on a Voyager-DE STR (Applied Biosystems) MALDI-TOF instrument at the Proteomics facility at SUNYSB in a linear mode.

### Ape1 fragment identification by MALDI-TOF and LC/MS

The full length APE1 and its 25 kDa fragment were digested in gel by trypsin and analyzed by MALDI-TOF and LC/MS. Voyager-DE *STR* (Applied Biosystems) MALDI-TOF instrument at the Proteomics facility at SUNYSB in a reflector mode was used and the matrix was Alpha-cyano-4-hydoxycinnamic Acid (CHCA).

### Docking studies

The screening used the AutoDock suite of programs [39]–[41]. The screening was driven by a set of scripts described in Mezei *et al* [42]. Models of lucanthone and hycanthone were generated with Marvin Sketch (figure S1) (ChemAxon, Budapest, Hungary), and the structures optimized with the semiempirical AM1 method, as implemented in Gaussian-03 [REF_G] – the script set referred to above includes utilities to create the Gaussian input and the extraction of the optimized coordinates. The terminal amine was protonated, as determined previously and the overall structures were in good agreement with the X-ray structure of hycanthone [43]. The protein structure was obtained from the Protein Data Bank (PDB ID: 2ISI). Assignment of atom types, and partial charges (using Gasteiger –Marsili method) and merging of non-polar hydrogens with their carbons was performed with AutoDockTools (http://mgltools.scripps.edu/downloads). The generation of the energy grids, the preparation of ligand files for docking and the actual docking was driven by a script that keeps a user-defined number of docking jobs running on different processors of our SGI cluster [42].

Dockings were based on a 126×106*126 grid, with grid spacing of 0.375 Å that targeted the hydrophobic pocket lined by hydrophobic amino acids Trp280, Phe266 and Leu282. The minimization resulting in docked poses was performed using Lamarckian genetic algorithm (LGA) and pseudo-Solis and Wets method. Each LGA job consisted of 200 runs with 270, 000 generations in each run and maximum number of energy evaluations of 2,000,000. After clustering of the docked poses by Autodock the program Dockres (URL: http://inka.mssm.edu/~mezei/dockres) sorted cluster representatives of the docked poses and extracted the list and coordinates of the top-scoring ones in complex with the target protein.

### Molecular dynamics (MD) simulations

The docking top-scoring poses APE1-lucanthone and APEI-hycanthone were solvated, using VMD [44], in a water box extending 10 Å beyond the edge of the complex in all directions, using the TIP3P water model [45]. The system was neutralized with chloride ions and consisted of a total of ∼36,100 atoms. MD calculations were performed with NAMD [46] using the AMBER99SB force field [47]. The ligand parameters were determined with ANTECHAMBER [48] using the General Amber Force Field (gaff) [49]. Partial atomic charges were determined with the AM1-BCC method. The systems were energy minimized with 10,000 steps of conjugate gradient energy minimization, followed by gradual heating from 0 to 310 K in 30 ps, and then maintained at constant temperature and pressure (1.01325 bar). The simulations were carried with periodic box conditions, with a 2 femptosecond time step, a uniform dielectric constant of 1, a 1–4 scaling value of 0.833333, a cutoff of non-bonded forces with a switching function starting at 10 Å and reaching 0 at 12 Å, PME with a tolerance of 10^−6^, and all bonds involving hydrogens constrained with the SHAKE algorithm. A production run was performed for 30 ns and the trajectories were analyzed with VMD.

### EMSA assay

In order to elucidate whether lucanthone and hycanthone could inhibit APE1 by interfering with APE1-DNA interaction through their DNA intercalation ability, we measured the DNA binding capacity of APE1 in the presence of lucanthone and hycanthone by incubating different concentrations of APE1 (10 nM and 25 nM) with 100 µM of lucanthone and/or hycanthone for 30 min and subsequently incubating the mixture with 25 nM of THF-containing substrate at 25°C in binding buffer (50 mM HEPES, pH 7.5, 150 mM NaCl, 0.1 mg/ml BSA, 0.5 mM EDTA and 1 mM DTT) for 30 min. THF stands for tetrahydrofuran that represents a synthetic abasic site. This is commonly inserted into an oligonucleotide substrate for measuring APE1 cleavage activity. 5′-incision of THF by APE1 results in a residue that mimics reduced 5′-deoxyribose phosphate group. Since THF group lacks the 1′-OH group, it blocks β-elimination that is required for mediating any dRP lyase activity. The sequence of the APE1 substrate with THF group is 5′CTGCAGCTGATGCGCFGTGCGGATCCGGTGC-3′ as described by Liu *et al* [50].

APE1-DNA complex was then separated from unbound substrate DNA by electrophoresis under native conditions in a 1% agarose-0.1% acrylamide gel at 4°C for 1.5 h as described previously [50]–[51].

## Results

### Lucanthone/hycanthone promotes APE1 cleavage

**Figure 1:**
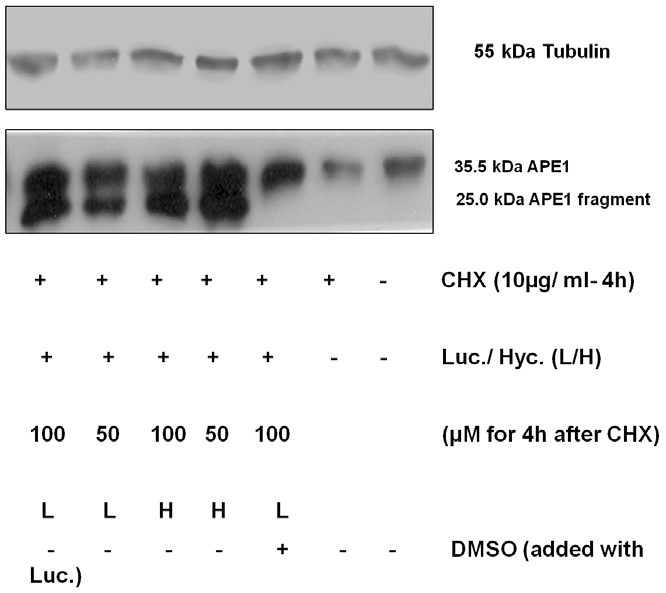
Lucanthone/Hycanthone promotes APE1 cleavage in presence of CHX and this cleavage is inhibited by 1% DMSO. Western blot of total cell extract from APE1-5 overexpresser clone pretreated with 10 µg/ml of cycloheximide for 4 h followed by 25–100 µM lucanthone/hycanthone for 2 h (12.5 mg of total cell protein loaded per lane).

Previously, we created an APE1 overexpressor glioma cell line, the U251 1–5 clone [52], to determine the contribution of APE1 to radio-resistance. Since past studies had shown that lucanthone inhibits DNA and RNA synthesis [53] [54] with or without affecting protein synthesis [53], we determined the effect of lucanthone on APE1 protein expression in the U251 1–5 APE1 overexpressor cell line pre-treated with 10 µg/ml cycloheximide (CHX) (a protein synthesis inhibitor) for 4 h. We found that de novo APE1 synthesis was not affected significantly, as seen by the near normal levels of intact APE1 in CHX treated cells (Figure 1). However, lucanthone and hycanthone were found to induce cleavage of APE1 as seen by the formation of a 25 kDa fragment.

**Figure 2:**
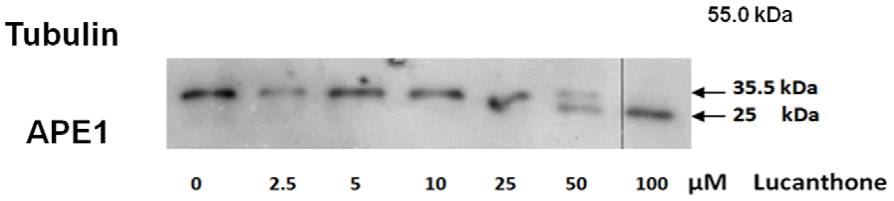
Lucanthone promotes APE1 cleavage in presence of protease inhibitor. Western blot of APE1 over expresser clone 5 pretreated with increasing (2.5–100 µM) concentration of lucanthone in presence of protease inhibitor cocktail for 2 h at 37°C (10 µg of total cell protein loaded per lane). An arrow indicates the corresponding increase in APE1 25 kDa fragment in last two lanes with a decrease in 35.5 kDa APE1 protein.

To begin to define the mechanism of cleavage, we treated whole cell extracts from the U251 1–5 APE1 overexpressor clone with increasing concentrations of lucanthone (2.5–100 µM) and examined APE1 protein stability in the presence of a protease inhibitor cocktail. We found that lucanthone at 50 and 100 µM caused APE1 cleavage and/or degradation as evidenced by a decrease in the full-length 35.5 kDa fragment and an increase in a ∼25 kDa fragment (Figure 2). These data suggested a direct effect of lucanthone on APE1 protein integrity, as effects on gene expression are not relevant in this paradigm. It is highly unlikely that the drug preparation was contaminated with a protease, as the compound was synthesized by organic methods *de novo* and handled to avoid any protease contamination. In addition, we found no evidence of non-specific degradation of other control proteins, such as tubulin and human NTH1 (human Nth was used as a DNA repair enzyme control, since like APE1, human Nth has a disordered N-terminus; data not shown).

**Figure 3:**
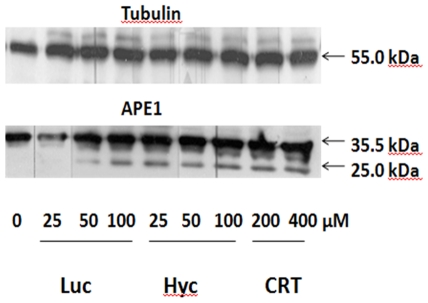
Lucanthone/Hycanthone promote APE1 cleavage at lower concentration than CRT. Western blot of APE1 over expresser clone 5 pretreated with increasing concentration of lucanthone (2.5–100 µM) and CRT0044876 (2.5–200 µM) in presence of protease inhibitor cocktail for 2 h at 37°C (10 µg of total cell protein loaded per lane). The corresponding decrease in 35.5 kDa APE1 protein band and increase in 25-kDa-degradation product is indicated by the arrows.

We next determined the effect of lucanthone, as well as CRT, a commercially available APE1 inhibitor modeled to bind at the hydrophobic site of the protein [13], on the stability of APE1 and tubulin in the presence of a protease inhibitor cocktail. As shown in Figure 3, we found that lucanthone caused cleavage of APE1 at 50 µM in U251 1–5 whole cell extracts, whereas for similar cleavage to occur with CRT, we needed to use 200 µM of the inhibitor. The tubulin protein was unaffected by lucanthone, but showed some shift in its migration at 200 µM CRT.

### Recombinant full length APE1 is also cleaved by lucanthone

**Figure 4:**
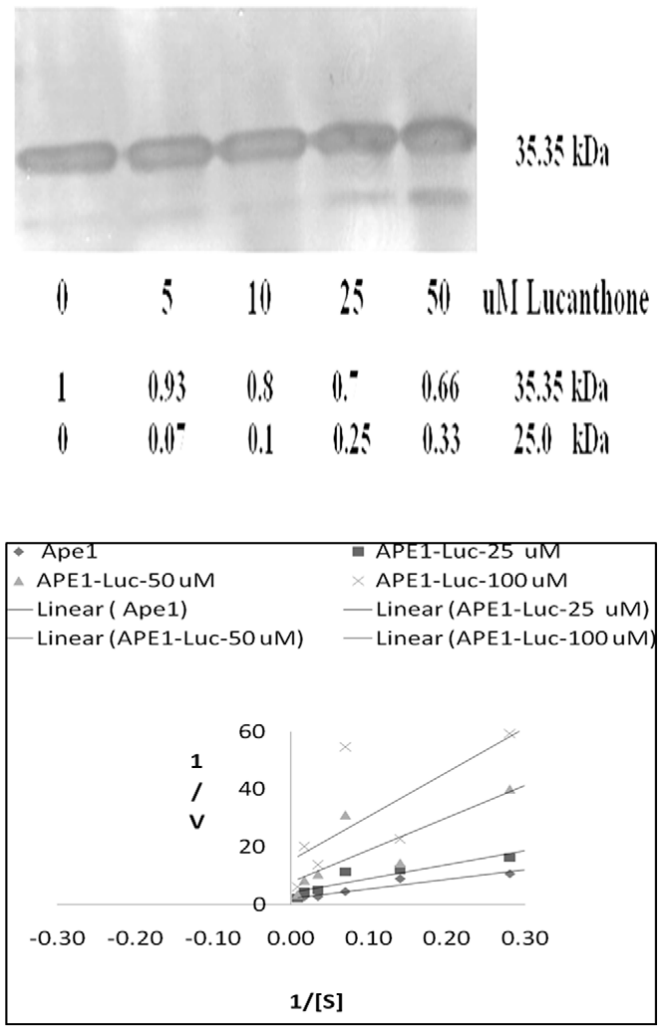
Lucanthone promotes APE1 cleavage *in vitro* by possible non-competitive binding. A. Recombinant APE1 protein (250 ng) treated with 10–50 µM lucanthone at 37°C for 2 h, the numbers in italic font represent fold change in APE1 and its 25 kDa fragment as measured by area analysis using Image J quantification program. B. Lineweaver-Burke plot for endonuclease assay determinations as detailed in Materials and Methods. Units of 1/v were min/fmoles of abasic sites incised and for 1/[S] was inverse of nM of depurinated plasmid DNA.

To further delineate the mechanism of lucanthone-induced cleavage, studies were performed with recombinant full length APE1 protein. When the recombinant protein was treated with 10–50 µM lucanthone at 37°C for 2 h, we observed an increase in the formation of the 25 kDa fragment and a corresponding decrease in AP endonuclease activity (Figure 4A and 4B). When we studied the kinetics of inhibition shown by lucanthone, we found that lucanthone appeared most likely to be a non-competitive inhibitor of APE1 (Figure 4B). When human Nth was treated with lucanthone, we did not observe any cleavage (data not shown), indicating specificity for APE1. These data imply that lucanthone directly binds to APE1.

**Figure 5:**
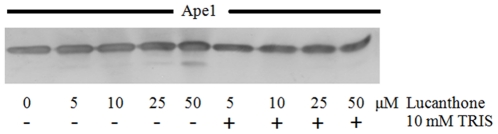
Lucanthone promotes APE1 cleavage in vitro which is inhibited by TRIS. Western blot of recombinant APE1 protein (250 ng) treated with 10–50 µM lucanthone at 37°C for 2 h in absence and presence of radical quencher, 10 mM Tris-HCl, pH 7.4.

**Figure 6:**
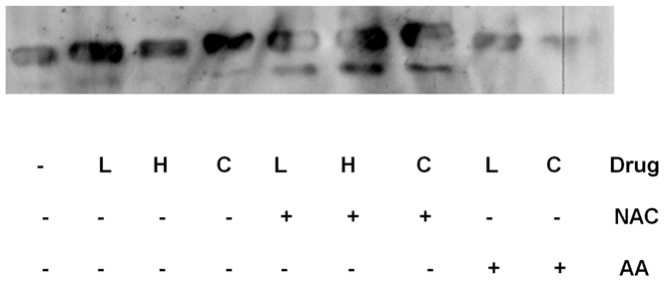
Thioxanthenones and CRT cleavage of APE1 is inhibited by Ascorbic acid but not by N-Acetyl cysteine. Western blot of recombinant APE1 protein (250 ng) treated with 100 µM of radical quenchers, NAC and Ascorbic acid and 100 µM of lucanthone (L)/hycanthone (H) or 200 µM of CRT (C) at 37°C for 2 h.

As we observed that lucanthone/hycanthone induced cleavage of APE1, we determined if this cleavage might be due to oxidative damage of the peptide bonds in the protein. We therefore performed an experiment in the presence of the short and long lived radical quenchers: 10 mM (final) tris(hydroxymethyl)aminomethane (TRIS), 100 µM ascorbic acid or 100 µM N-acetyl cysteine (for purified APE1 protein) and 1% DMSO (for APE1 protein from overexpressor cell extracts), and found that lucanthone-induced cleavage of APE1 was significantly inhibited (Figure 5 and Figure 6). As lucanthone is made of a strong hydrogen bonding acid, a secondary amine and two proton bonding bases (the carbonyl and tertiary amine substituent) and is known to possess a strong intramolecular amino carbonyl hydrogen bond [32], which can possibly attack the amide linkage in APE1, this may be one of the ways it can affect APE1 integrity.

### APE1 directly interacts with lucanthone and hycanthone

**Figure 7:**
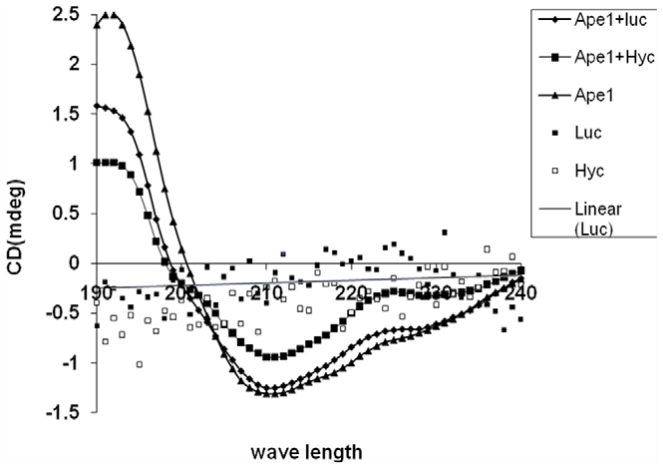
Lucanthone and hycanthone directly alter APE1 conformation. CD spectra of APE1 in presence of lucanthone and hycanthone as described in materials and methods, which was analyzed by Dichroweb program CDSSTR.

CD spectral studies revealed considerable conformational changes in APE1 in the presence of either lucanthone or hycanthone, indicating a direct physical interaction between the protein and small molecule (Figure 7). In particular, a significant change in the helical portion of the protein was evident, as seen by a decrease in the average helical length per segment (Table 1).

**Table 1:** Changes in APE1 conformation in presence of lucanthone and hycanthone.

Sample	Helix 1	Helix 2	Av. helix length per segment	Strand 1	Strand 2	Av. strand length per segment	Turns	Unordered
**APE1**	0.01	0.069	4.512	0.261	0.140	5.791	0.126	0.394
**APE1 +Luc**	0.00	0.071	4.053	0.262	0.140	5.762	0.125	0.403
**APE1 +Hyc**	0.00	0.069	4.020	0.267	0.141	5.799	0.124	0.399

Differences in CD conformation in presence of Lucanthone and Hycanthone. Data in Table 1 represent the CD analysis of APE1 in presence of lucanthone and hycanthone (Figure 7) which are shown as changes in α helix, β sheet and unordered conformation of APE1 protein. APE1 protein (5 mg/ml), 50 µl (250 µg) in APE1 buffer (50 mM HEPES, 150 mM KCl, 5 mM MgCl_2_), was mixed with lucanthone/hycanthone (1 mg/ml), 50 µl (50 µg), incubated at 37°C for 60 min and far UV-CD spectra were recorded at NSLS U11 beam line at BNL. These data are representation of three independent repeats.

**Figure 8:**
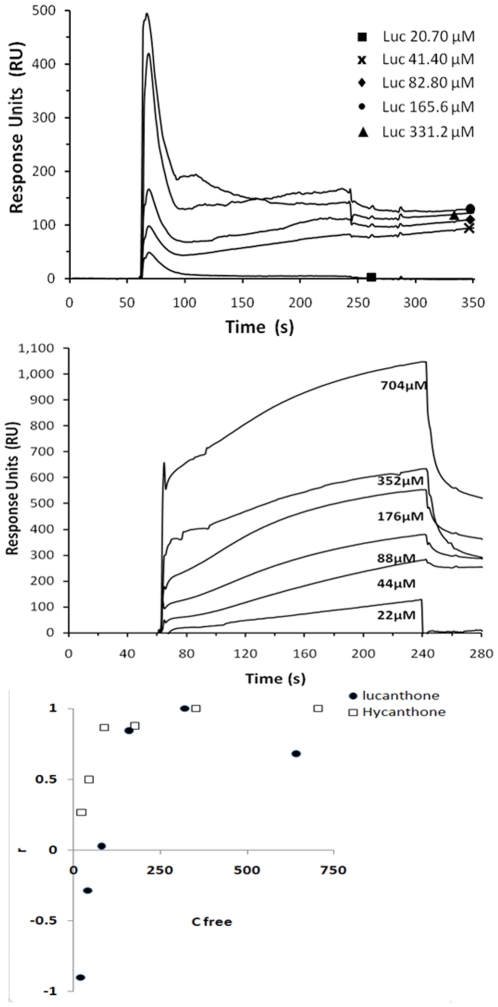
APE1 binds directly with lucanthone and hycanthone with different affinities. APE1 protein (100 µg) (ligand) was immobilized on carboxymethyl-5 (CM-5) chip by amine coupling according to manufacturer's instructions. Hycanthone (top figure) (analyte) and lucanthone (analyte) (lower figure) (analyte) at different concentrations (as shown as numbers representing µM values) were tested for binding to APE1 on BAICORE 2000 SPR measurement system available at SUNYSB proteomics core facility. The observed maximum response (RU) was determined by direct curve fitting of the obtained data assuming a 1∶1 interaction model. The third sub figure shows plot of r (RU/RU_max_) versus Cfree. Binding studies were carried out 3 times and data presented are representative of those 3 separate experiments.

To further characterize the apparent molecular interaction between APE1 and lucanthone/hycanthone, direct binding studies were undertaken using measurements of surface plasmon resonance relative response units (RU) on a BIACORE 2000, which can determine the binding constant, as well as the rates and the stoichiometry of the reaction [48]. In these experiments, a very good binding response was observed between APE1 and hycanthone (Figure 8, top), with weaker binding to lucanthone (Figure 8, bottom). The association phase was weak for lucanthone (see Figure 8, bottom and Table 2), whereas for hycanthone there was a much stronger association, with the k_a_ and k_d_ of full length APE1 being about 10-fold higher and 100-fold lower, respectively, for hycanthone. The spike in RU value seen with lucanthone may be due to the initial injection coupled with poor binding, which causes an uneven plateau formation (these seen also figure S2); when higher concentration was used, a clear plateau was seen (figure S2). The negative values seen for lower concentrations of lucanthone are most probably due to very poor interaction of lucanthone with APE1 under BIACORE binding conditions, which when compared to buffer control resulted in no surface plasmon resonance. As the drugs were dissolved in distilled water, we did not see any precipitation until 1 mM. The APE1: lucanthone/hycanthone stochiometry appeared to be close to 1 for hycanthone, but may have been lower for lucanthone. “r” versus Cfree plot was done to determine the differences in affinity of lucanthone and hycanthone and it was clear that hycanthone had much higher binding affinity as compared to lucanthone.

**Table 2:** Kinetics of lucanthone and hycanthone binding to APE1.

Inhibitor	Conc (µM)	RU_ligand_	RU_max_	k_a_ (1/Ms)	k_d_ (1/s)	KD (nM)
LUC	20	−82	91	878	7.76×10^−5^	89
	40	−26				
	80	2.5				
	160	77				
	320	94				
	640	62				
HYC	22	24	90	90	8.9×10^−7^	10
	44	45				
	88	78				
	176	79				
	352	90				
	704	118				

Hycanthone shows higher binding affinity as compared to Lucanthone. Data in Table 1 show affinity analysis of lucanthone (LUC) and hycanthone (HYC) binding to APE1 immobilized on CM5 chip (Figure 6A and 6B) using BIAsimulation software available at the Biacore facility at SUNYSB.

RU = Resonance units are measure of changes in refractive index; k_a_ = association constant; k_d_ = dissociation constant and KD = Affinity constant. These data are representation of two independent repeats.

### Lucanthone Cleavage site identification with LC/MS

**Figure 9:**
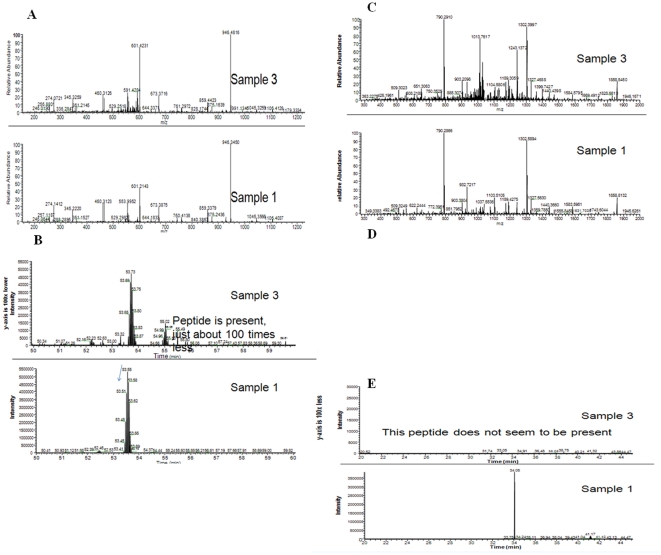
Identification of an approximate lucanthone cleavage site in APE1. LC/MS/MS identification of APE1 fragment after treatment with 100 µM of lucanthone for 2 h at 37°C. The data were analyzed with Inspect. A). Peptide aa64–73 analysis of 35.5 Kda (Sample 1) and 25 kDa (Sample 3). B). Elution profile of peptide aa64–73 in sample 1 and 3. C). Peptide aa282–299 analysis of 35.5 Kda (Sample 1) and 25 kDa (Sample 3). D). Peptide aa53–63 analysis of 35.5 Kda (Sample 1) and 25 kDa (Sample 3). B). Elution profile of peptide aa53–63 in sample 1 and 3.

Preliminary data with sequence identification of APE1 protein (sample 1) and its 25 kDa fragment (sample 3) found the cleavage site to be between amino acid (aa) 53–63 resulting in about a 20 kDa fragment as shown in Figure 9. Peptide analysis of aa 64–73 reveals that sample 3 begins at aa 64 and that this peptide is about 100 times lower in concentration in sample 3. Peptide analysis of aa 282–299 reveals that sample 3 ends after aa 299. Peptide aa 53–63 is not present in sample 3. It appears that the degraded protein (sample 3) has peptide present from aa 64 until at least aa 299. Peptide aa 53–63 is not present, suggesting that the cut/degradation is in the vicinity of aa 53–63. The precise N-terminal sequence identification is needed to get the exact cleavage site and studies are under way to determine that site.

### Degradation of APE1 by lucanthone and hycanthone

**Figure 10:**
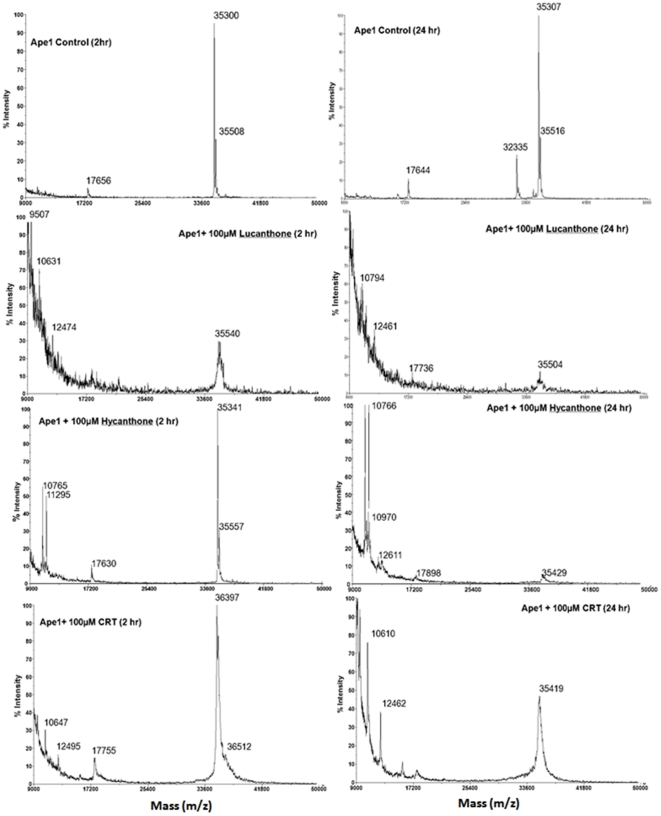
Lucanthone, Hycanthone and CRT cause APE1 degradation. Mass spectroscopic (MALDI-TOF) analysis of APE1 in presence of lucanthone and CRT as described in materials and methods. APE1 (100 µM) was treated with 100 µM lucanthone and CRT for 2 h and 24 h and changes in its mass was determined as described in Materials & Methods.

MALDI TOF analysis was undertaken for samples analyzed previously (SDS-PAGE/western blot; see earlier) to determine the nature of the APE1 protein fragments in the presence of lucanthone, hycanthone, or CRT at 37°C for 2 or 24 h. These studies revealed steady degradation of APE1 into smaller fragments (Figure 10) by lucanthone with time; the signal of the parent 35.5 kDa peak was also significantly reduced, consistent with APE1 breakdown. CRT treatment at the same concentration resulted in much less cleavage of APE1, with the 35.5 kDa peak remaining almost intact, particularly at 2 h, and only a couple of smaller fragments of 10, 12 and 17 kDa appearing at 24 h. Hycanthone showed cleavage of APE1 as evidenced mainly by 10, 11 and 17 kDa peaks, without the other smaller fragments seen with lucanthone. By 24 h at 37°C, the full length APE1 peak was almost completely degraded in the lucanthone/hycanthone treated samples, whereas there was still an intact 35.5 kDa peak with CRT (Figure 10). Even though we saw cleavage of recombinant APE1, we did not see the 25 kDa fragment observed in the extract studies or recombinant protein experiments described above (Figures 1 and 4). However, as we did see fragments of 10–17 kDa on MALDI, it is possible that the 25 kDa fragment degraded into these smaller fragments during the analysis.

### Molecular docking of lucanthone and hycanthone at the hydrophobic site of APE1

**Figure 11:**
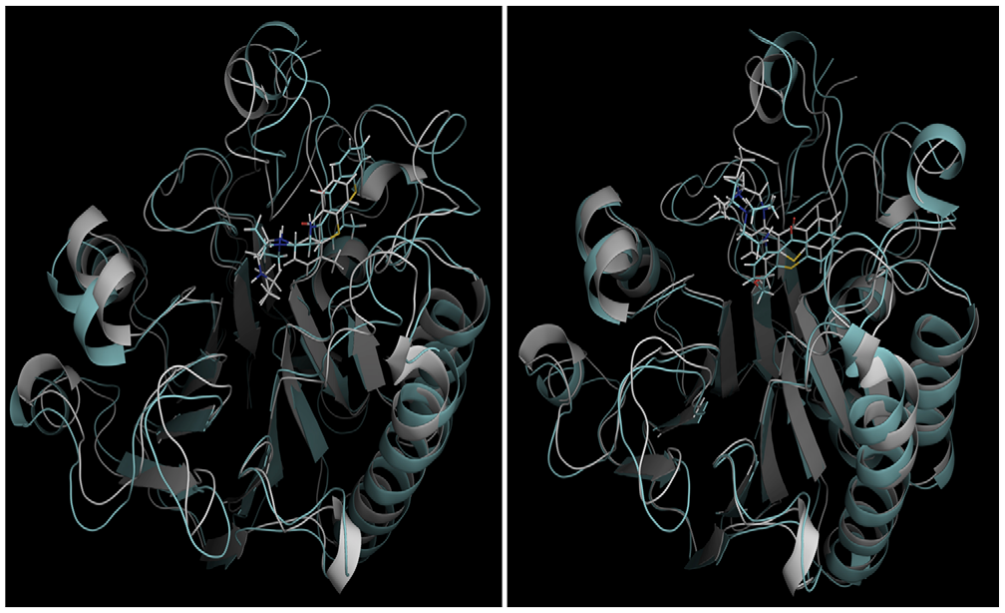
Lucanthone and hycanthone docks at hydrophobic site in APE1. A) The lowest energy pose of docked APE1/lucanthone (gray) superposes with the structure of the complex post 30 ns of MD simulation with an r.m.s.d. of 1.8. Å. B) docked APE1/hycanhone (gray) superposes with the structure of this complex post 30 ns of molecular dynamics simulation with an r.m.s.d. of 1.7. Å.

**Figure 12:**
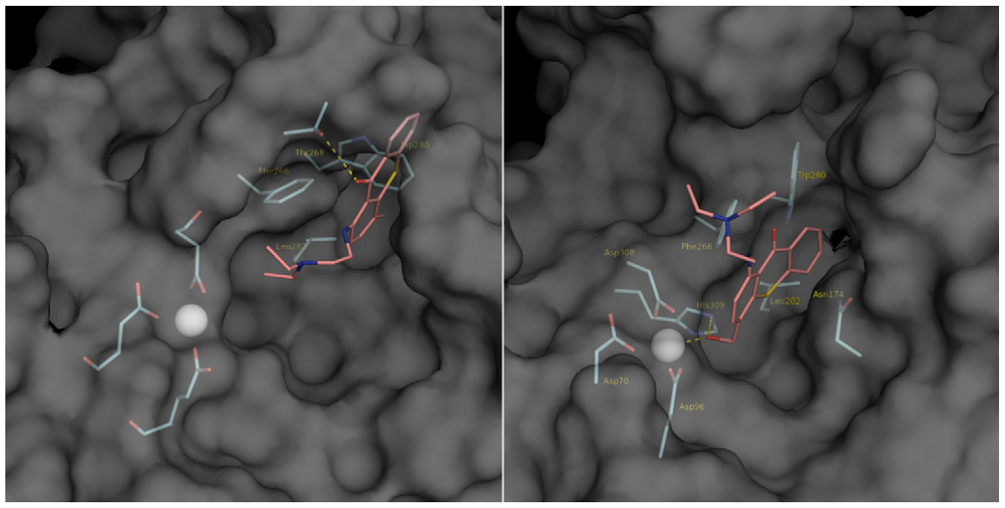
Molecular Dynamics simulation of APE1-bound Lucanthone and Hycanthone. A: lucanthone (magenta sticks) binds in the hydrophobic pocket of Ape1, represented by its solvent accessible surface. Ape1 interacts with lucanthone mainly via apolar contacts with W280, pi-stacking with F266, and M270. Additionally, a hydrogen bond forms between the carbonyl oxygen and T268. B) Hycanthone (magenta sticks) binds in the DNA groove/hydrophobic pocket of APE1. In addition to apolar contacts and pi-stacking with F266, it forms a hydrogen bond with His309 and coordinates with the APE1-bound magnesium cation (represented as gray sphere).

To examine possible binding modes of lucanthone and hycanthone with APE1, in silico molecular docking was performed with the AutoDock suite of programs (Figure 11). To account for the flexibility of structural elements and aa side chains, the top-scoring pose of each ligand (based on the energy evaluation of AutoDock and clustering of the poses) was fully solvated and submitted to a 30 ns MD simulation. After an initial equilibration in which the protein side chains and the ligand re-adjusted their positions, the ligands were in a stable conformation for up to the 30 ns simulated, undergoing an average rmsd <2 Å for the second half of the simulation (data not shown). Overall, the docked complexes of APE1-lucanthone and APE1-hycanthone superpose with the MD simulated structures with an r.m.s.d. (root mean square deviation) of 1.8 Å/1.7 Å (Figure 12A and B). Lucanthone binds deep in the hydrophobic pocket (Figure 12A), interacting with the protein mainly via apolar contacts. The phenyl group of lucanthone forms a parallel-displaced pi-stacking interaction to Phe266, while the carbonyl group forms a transient hydrogen bond with Thr268, with a average distance of 3.4 Å between donor and acceptor throughout the simulation. The long flexible side chain of the tertiary amine of lucanthone extended to the DNA binding groove. The hycanthone binding site was shifted towards the DNA binding groove and the unsubstituted ring was buried in the hydrophobic pocket of the protein, in a position to form a parallel pi-stacking interaction with Phe266. The hydroxyl group of hycanthone formed a stable hydrogen bond with His309, while the oxygen atom was stably coordinated with the APE1 bound magnesium cation (Figure 12B). The flexible side chain of hycanthone extended into the solvent and made no protein contacts.

### Hydrophobic site mutants do not undergo dramatic conformational changes in the presence of lucanthone

**Figure 13:**
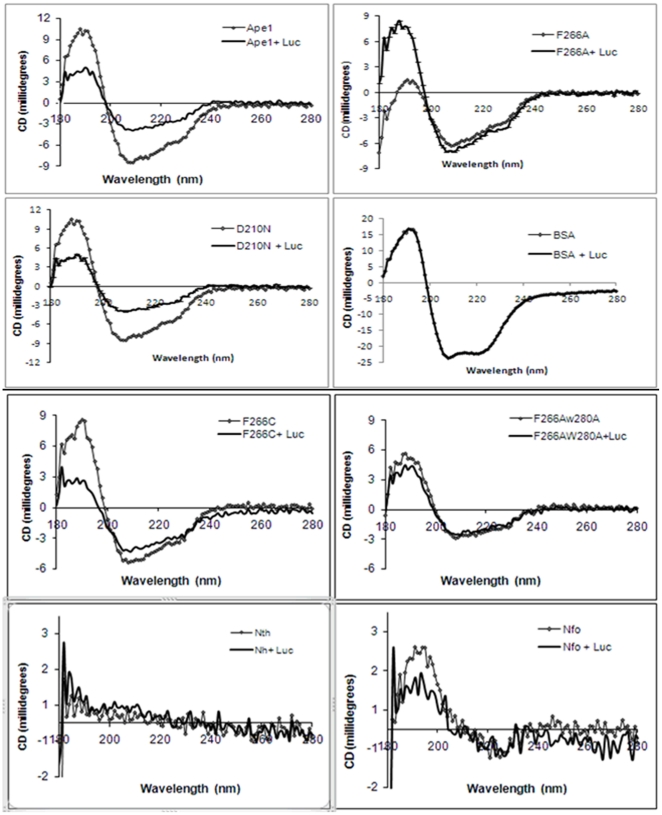
Lucanthone does not alter conformation of double hydrophobic site APE1 mutant, human Nth and bacterial Nfo proteins. CD spectra of APE1 and its mutants in presence of lucanthone. APE1- hydrophobic site mutant proteins F266A/C, F266A/W280A, active site mutant D210N and non-related BSA protein or human Nth or E.coli Nfo(10 mg/ml), 50 µl (500 µg) (14 µM) in APE1 buffer (50 mM HEPES, 150 mM KCl, 5 mM MgCl_2_), was mixed with lucanthone (1 mg/ml), 50 µl (50 µg) (140 µM), incubated at 37°C for 60 min and far UV-CD spectra with specifications.

In light of the docking studies above, and since previous APE1 inhibitors (e.g. CRT and L-DOPA) were postulated to interact in a similar manner with the protein [13], [55], we determined whether mutating a single hydrophobic site residue, Phe266 to Ala/Cys (F266A or F266C), or mutating two hydrophobic residues, Phe266Ala/Trp280Ala (F266A/W280A), would prevent the lucanthone-induced conformational changes in APE1. Bovine serum albumin (BSA) was included as a non-specific protein control and the active site APE1 mutant Asp210Arg (D210N) was included as a non-hydrophobic site control, presenting the same binding ability but failing to cleave the DNA. Human NTH1 and *E.coli* endonuclease IV (Nfo) were used as other DNA repair enzyme controls. As shown in Figure 13, lucanthone was able to induce conformational changes in the wild type and D210N active site mutant APE1 proteins, but altered the hydrophobic site mutant protein F266A or F266C to a lesser extent, while inducing almost no conformational change in the double mutant F266A/W280A. Lucanthone also caused little conformational changes on human Nth and *E.coli* Nfo. These data indicate the importance of the hydrophobic site residues F266 and W280 in binding the small molecule inhibitor, and support the binding mechanism proposed by molecular modeling.

### Hydrophobic site mutants are not cleaved by lucanthone and show lower inhibition of their endonuclease activity in the presence of lucanthone

**Figure 14:**
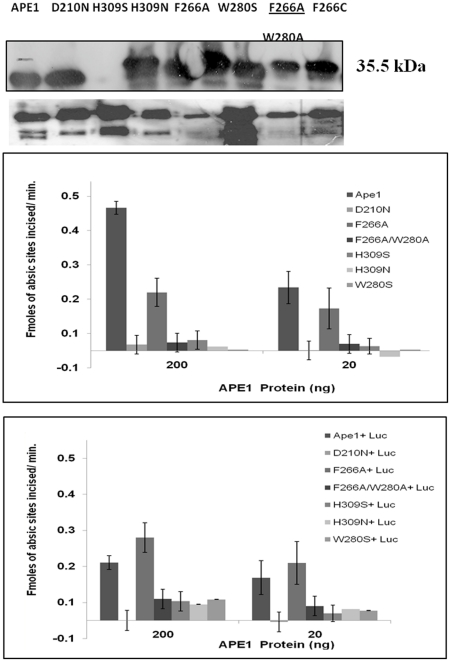
Lucanthone causes cleavage of APE1 with intact hydrophobic site. (A). Western blot of recombinant and mutant APE1 proteins (upper panel) treated with 100 µM lucanthone (lower panel) at 37°C for 2 h. (B). Endonuclease activity inhibition of wild type and F266A mutant of APE1 in presence of 100 µM of lucanthone for 2 h at 37°C.

As we found that F266A, F266C or F266A/W280A proteins did not undergo a significant conformational change in the presence of lucanthone, we hypothesized that due to impaired binding, lucanthone would show a lesser effect on protein cleavage and endonuclease incision efficiency [34]. As seen in Figure 14A and B, all the hydrophobic site mutants, except W280S, did not undergo cleavage (this W280S mutant also showed conformational change, figure S3), whereas the active site mutant D210N, His309Ser (H309S) and His309Arg (H309N) were degraded. In addition, lucanthone caused a corresponding inhibition of the endonuclease activity of wild type, but no inhibition was detected for the already reduced activity of the single mutants (F266A or W280S), whereas the effect on endonuclease activity of the double hydrophobic mutant's endonuclease activity could not be detected, as this double mutant (F266A/W280A) had very reduced activity as reported earlier [56].

### Lucanthone and hycanthone only marginally inhibit the DNA binding capacity of APE1

**Figure 15:**
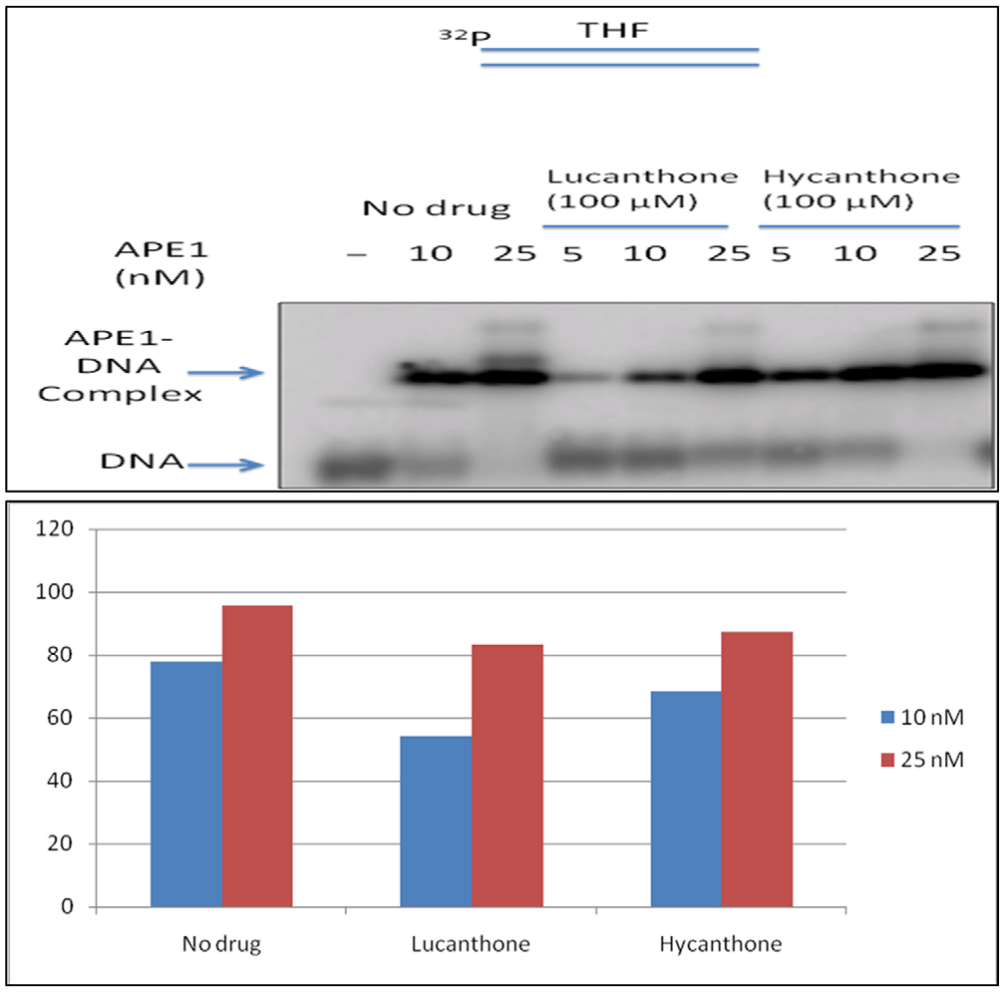
Lucanthone and Hycanthone do not significantly affect the DNA binding capacity of APE1. Gel mobility shift assay for determining the effect of lucanthone and hycanthone on DNA binding capacity of APE1. APE1 at 10 nM and 25 nM was mixed with 100 µM lucanthone or 100 µM hycanthone in the buffer that contained 50 mM HEPES, 150 mM KCl, 0.1 mg/ml BSA, 0.5 mM EDTA and 1 mM DTT. The mixture was incubated at room temperature for 30 min. Subsequently, 10 nM radiolabeled substrate that contained an abasic site was added in the mixture and incubated with the enzyme for additional 30 min to allow binding of APE1 to the substrate. 8 µl binding mixture was subject to electrophoresis at 4°C, 100 V for 1.5 h in a 1% agarose–0.1% acrylamide gel. The gel was then dried on DE81 paper, and APE1-DNA complex was detected by phosphorimager as described previously [52].

As lucanthone and hycanthone are well known DNA intercalators, preferentially intercalating at AT rich sites in DNA, we determined whether inhibition of APE1 endonuclease activity was due to compound-DNA interactions, which may block APE1 access to the abasic substrate. The results from a gel mobility shift assay demonstrated that both lucanthone and hycanthone, which were pre-incubated with the protein prior to incubation with the substrate DNA, only marginally inhibited or failed to inhibit APE1 DNA binding capacity as shown in Figure 15.

## Discussion

Our recent studies [10] showed that APE1 plays a significant role in the survival of the glioblastoma cell line U87 as compared to U251. Indeed, we found a direct correlation between the level of APE1 expression and radio-resistance. In addition to our APE1 overexpressor studies with the radio-sensitive cell line U251, we also showed that suppressing APE1 protein levels with the small molecule inhibitor lucanthone caused radio-sensitization. Lucanthone was chosen as an APE1 inhibitor for several reasons: It is in a Phase I clinical trial for tumor radiotherapy, its side effects are almost negligible, and it can inhibit APE1 endonuclease activity without affecting its redox function. However, as lucanthone has good DNA intercalation ability, we hypothesized that the final outcome of APE1 inhibition might be due to a fine balance between direct and indirect effects. Thus, we sought to elucidate the mechanism of APE1 endonuclease inhibition by lucanthone.

As APE1 has a well-characterized hydrophobic site within its active site, we hypothesized those small hydrophobic molecules like lucanthone may stack in this site through van der Waals interactions and thus alter the active site causing repair endonuclease inhibition. When we treated U251 1–5 glioma cells with increasing concentrations of lucanthone, we observed a concentration-dependent decrease in the normal 35.5 kDa APE1 protein band, along with an increase in a 25 kDa fragment. Since this fragmentation was seen in the presence of protease inhibitors, it is not likely the result of enzyme-mediated proteolysis, but may occur due to a direct effect of lucanthone on the APE1 protein. We reproduced the finding of lucanthone induced APE1 degradation with purified recombinant protein, indicating that the cell machinery was not required for the cleavage event. MALDI TOF analysis of APE1 protein treated with lucanthone or hycanthone revealed a significant degradation of the 35.5 kDa peak and lower molecular weight species, although the 25 kDa fragment could not be found. This observation may suggest that the 25 kDa fragment is further degraded into smaller fragments. Sequencing of the 25 kDa APE1 fragment by MALDI TOF indicated the approximate cleavage site to be between aa 53–63. Our finding that short and long lived free radical quenchers, such as TRIS, ascorbic acid and DMSO, inhibit this degradation implies that protein fragmentation may be facilitated through a free radical mediated peptide bond cleavage reaction. The lack of inhibition of this cleavage by N-acetyl cysteine may indicate that a higher concentration of this free radical quencher may be needed or that thioxanthenones are able to render this quencher ineffective.

Our molecular docking, biochemical and biophysical studies, including those using various hydrophobic site and active site mutant APE1 proteins, support our hypothesis that lucanthone docks at the hydrophobic site. Furthermore, another APE1 inhibitor study found that several potent APE1 inhibitors contain two negatively charged ionizable groups separated by a hydrophobic core [57], features characteristic in lucanthone/hycanthone. As recently shown for the phenyl ring of the APE1 inhibitor, 6-hydroxy-DL-DOPA [55], the tri phenyl ring of lucanthone could form a pi stacking interaction to Phe266. Since our binding studies revealed that the K_a_ for hycanthone is about 8-fold higher than lucanthone, additional interactions between the former compound and the APE1 active site can be implied. Indeed, molecular docking revealed that in addition to key non-covalent van der Waals interactions seen with lucanthone, the extra hydroxyl groups of hycanthone can hydrogen bond with His309 and coordinate the APE1 bound magnesium cation. Our experimental studies found that lucanthone exhibited a lesser effect on the conformational status of the APE1 single hydrophobic site mutants F266A and F266C, and almost no effect on the double hydrophobic mutant F266A/W280A, consistent with no cleavage or significant effect on the endonuclease activity of these mutants (Figure 12A and B). However, surprisingly, we did see significant cleavage with W280S indicating that replacing the tryptophan residue with a serine did not alter the lucanthone induced cleavage. This is in stark contrast to the dramatic inhibition observed when phenylalanine was replaced by an alanine or cytosine, presumably reflecting lucanthone being able to bind the phenylalanine residue at 266 (as well as leucine at 282). Apparently F266 is the more critical residue involved in lucanthone binding.

We used another APE1 specific inhibitor CRT0044876 as a control, as it was modeled to bind to the hydrophobic site [13]. However, the effects of this inhibitor are unclear, as recent data on CRT have shown that its specificity for APE1 is controversial [58]–[60]. Nonetheless, in our cell system, CRT could inhibit APE1 protein at high concentrations (200 µM), as opposed to the inhibition we observed by lucanthone at 50 µM. The MALDI analysis also revealed clear degradation of APE1 in the presence of 100 µM lucanthone by 2 h, whereas at the same concentration, CRT0044876 did not cause degradation of APE1 until 24 h. These studies indicate that lucanthone and hycanthone are more potent APE1 inhibitors than CRT0044876.

As lucanthone and hycanthone were first used as DNA intercalators with good anti-tumor activity, another aspect of the inhibition of APE1 endonuclease activity likely involves an indirect effect. For instance, like the indirect effect seen for lucanthone or IA-5 on Topoisomerase II [61], the lucanthone–DNA intercalation may cause a distortion in DNA, leading to an impaired recognition of an abasic site by APE1. However, it is important to note that researchers have found no quantitative correlation between the ability of thioxanthenones to bind DNA and their antitumor ability [62], indicating that DNA intercalation is not sufficient for antitumor activity. These studies therefore indicate an important role for other macromolecules like proteins, such as DNA repair enzymes and accessory factors like HMGB1, in the potency of these compounds. Since the DNA binding ability of APE1 was only marginally altered by lucanthone and not by hycanthone, our data indicate that the effect of lucanthone is more likely due to its direct effect on the enzymology of APE1. Although lucanthone induced cytotoxicity has been attributed more to inhibition of Topo II [63], our present study, along with the past biochemical and cellular effects reported, indicate that APE1 is likely an important biological target. We therefore believe that APE1 may be developed as a biomarker for lucanthone-based treatment efficacy and that these studies provide a molecular framework for the design of more efficacious and clinically safe thioxanthenones.

## Supporting Information

Figure S1 **Lucanthone (left) and Hycanthone (right).** The Molecular structure was generated with Marvin Sketch.
(TIF)

Figure S2 **Lucanthone binding with APE1 reached saturation at higher concentration.** APE1 protein (100 µg) (ligand) was immobilized on carboxymethyl-5 (CM-5) chip by amine coupling according to manufacturer's instructions. lucanthone (analyte) with higher concentration (20–1325 µM) was tested for binding to APE1 on BAICORE 2000 SPR measurement system available at SUNYSB proteomics core facility.
(TIF)

Figure S3 **CD spectra of APE1 and its mutant W280S in presence of lucanthone.** APE1/W280 S (10 mg/ml), 50 µl (500 µg) (14 µM) in APE1 buffer (50 mM HEPES, 150 mM KCl, 5 mM MgCl_2_), was mixed with lucanthone (1 mg/ml), 50 µl (50 µg) (140 µM), incubated at 37°C for 60 min and far UV-CD spectra with specifications taken as described previously.
(TIF)
