# Supplementary figures and images for: Lucanthone and Its Derivative Hycanthone Inhibit Apurinic Endonuclease-1 (APE1) by Direct Protein Binding
Source: PLoS One. 2011 Sep 15;6(9):e23679. doi: 10.1371/journal.pone.0023679 (PMC3174134; doi:10.1371/journal.pone.0023679)

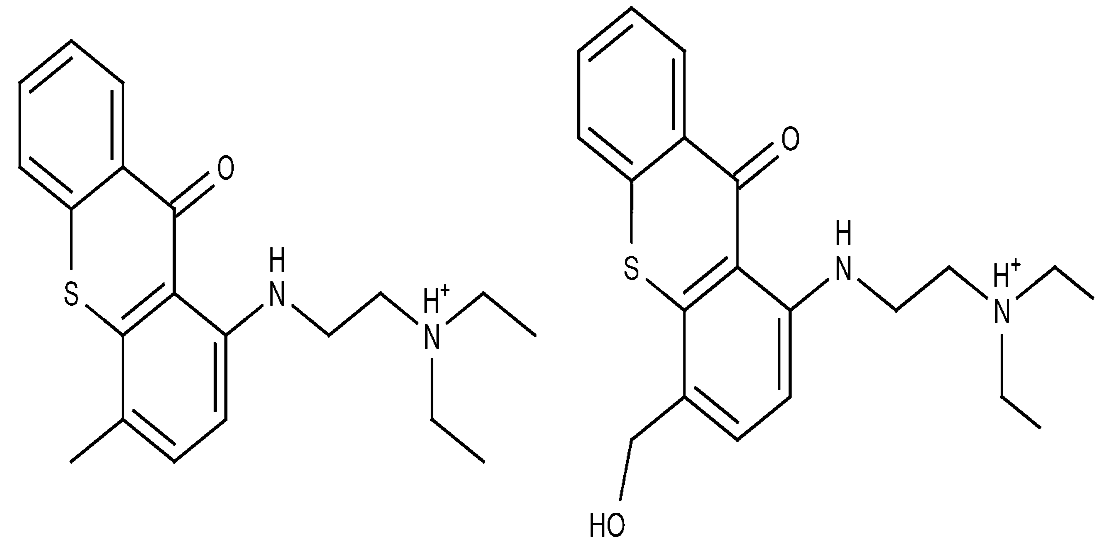

Supplement: Figure S1 — Lucanthone (left) and Hycanthone (right). The Molecular structure was generated with Marvin Sketch. (TIF) [file pone.0023679.s001.tif]

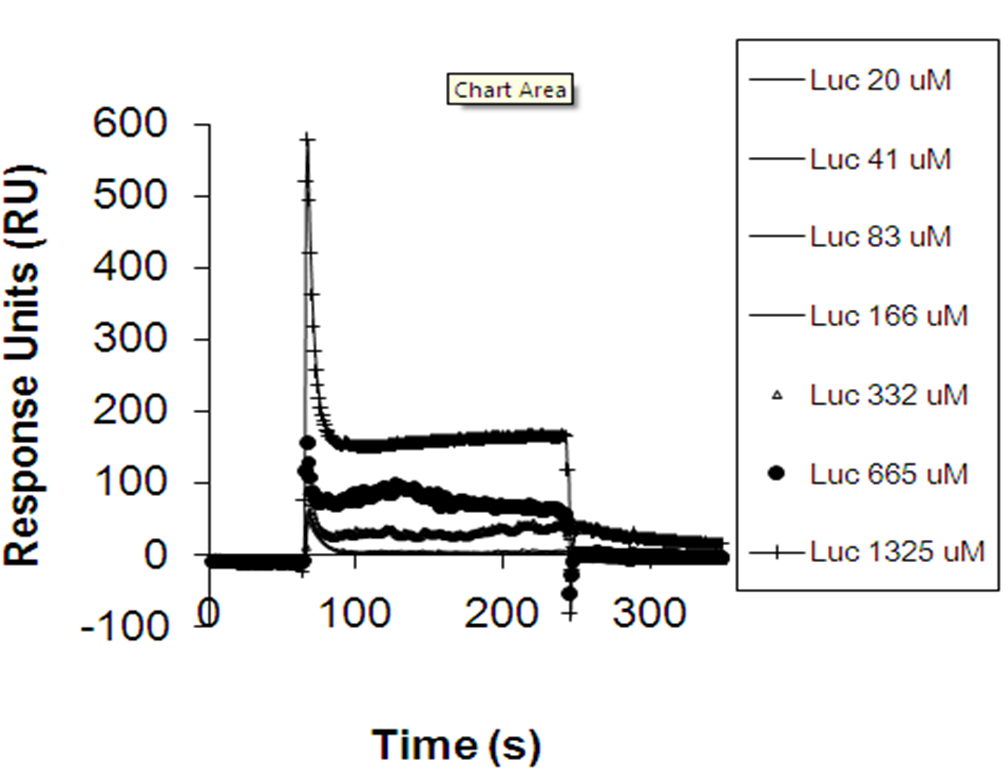

Supplement: Figure S2 — Lucanthone binding with APE1 reached saturation at higher concentration. APE1 protein (100 µg) (ligand) was immobilized on carboxymethyl-5 (CM-5) chip by amine coupling according to manufacturer's instructions. lucanthone (analyte) with higher concentration (20–1325 µM) was tested for binding to APE1 on BAICORE 2000 SPR measurement system available at SUNYSB proteomics core facility. (TIF) [file pone.0023679.s002.tif]

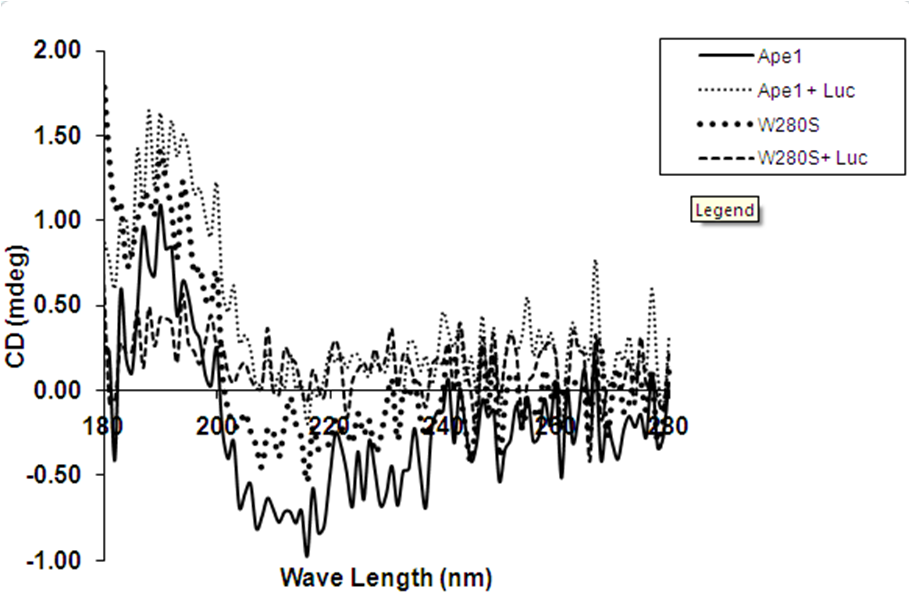

Supplement: Figure S3 — CD spectra of APE1 and its mutant W280S in presence of lucanthone. APE1/W280 S (10 mg/ml), 50 µl (500 µg) (14 µM) in APE1 buffer (50 mM HEPES, 150 mM KCl, 5 mM MgCl2), was mixed with lucanthone (1 mg/ml), 50 µl (50 µg) (140 µM), incubated at 37°C for 60 min and far UV-CD spectra with specifications taken as described previously. (TIF) [file pone.0023679.s003.tif]
